# Nutrient Transporter Expression in the Jejunum in Relation to Body Mass Index in Patients Undergoing Bariatric Surgery

**DOI:** 10.3390/nu8110683

**Published:** 2016-10-29

**Authors:** Brian A. Irving, G. Craig Wood, Peter N. Bennotti, Ellappan Babu, Abhishek Deshpande, Michelle R. Lent, Anthony Petrick, Jon Gabrielsen, William Strodel, Glenn S. Gerhard, Christopher D. Still, Vadivel Ganapathy, David D. K. Rolston

**Affiliations:** 1 Obesity Institute, Geisinger Health System, 100 N Academy Ave, Danville, PA 17822, USA; cwood@geisinger.edu (G.C.W.); pbenotti64@gmail.com (P.N.B.); mrlent@geisinger.edu (M.R.L.); gsgerhard@temple.edu (G.S.G.); cstill@geisinger.edu (C.D.S.); ddrolston@geisinger.edu (D.D.K.R.); 2 Department of Cell Biology and Biochemistry, Texas Tech University Health Sciences Center, Lubbock, TX 79430, USA; babu.ellappan@ttuhsc.edu (E.B.); vadivel.ganapathy@ttuhsc.edu (V.G.); 3 Medicine Institute, Cleveland Clinic Foundation, 9500 Euclid Avenue, Cleveland, OH 44195, USA; abhishekdp@gmail.com; 4 Surgery, Geisinger Health System, 100 N Academy Ave, Danville, PA 17822, USA; atpetrick@geisinger.edu (A.P.); jdgabrielsen@geisinger.edu (J.G.); westrodel@geisinger.edu (W.S.); 5 Department of Medical Genetics and Molecular Biochemistry, Lewis Katz School of Medicine, Temple University, Medicine Education & Research Building 3500 N. Broad St., Philadelphia, PA 19140, USA; 6 Internal Medicine, Geisinger Health System, 100 N Academy Ave, Danville, PA 17822, USA

**Keywords:** obesity, morbid obesity, nutrient transporters, nutrient absorption, gastrointestinal tract

## Abstract

Nutrient tranters (NT) facilitate nutrient absorption and contribute to the regulation of circulating nutrients. In this cross-sectional study, we determined the associations between the level of obesity; mRNA abundance for NTs; and serum concentrations of amino acids, short-chain fatty acids, and glucose in patients with morbid obesity undergoing a Roux-en-Y gastric bypass. Proximal jejunal samples were obtained at the time of surgery from 42 patients (90% female, age = 42.6 ± 11.9 years, pre-operative body mass index (BMI) = 55.5 ± 11.3 kg/m^2^) undergoing a Roux-en-Y gastric bypass. RNA was extracted from the jejunal mucosa and quantitative real-time–PCR was performed for the NTs studied. BMI negatively correlated with jejunal mRNA abundance of the amino acid NTs *TauT* (*r* = −0.625, *p* < 0.0001), *ASCT2* (*r* = −0.320, *p* = 0.039), *LAT1* (*r* = −0.304, *p* = 0.05). BMI positively correlated with jejunal mRNA abundance of the lactate/short-chain fatty acid NT *SMCT1* (*r* = 0.543, *p* = 0.0002). Serum concentrations of the short-chain fatty acids, butyric, valeric, and isocaproic acid correlated positively with BMI (*n* = 30) (*r* = 0.45, *r* = 0.44, *r* = 0.36, *p* ≤ 0.05; respectively). Lower jejunal mRNA abundance for the amino acid NTs *TauT*, *ASCT2*, and *LAT1* could protect against further obesity-related elevations in circulating amino acids. The positive correlation between BMI and the jejunal mRNA abundance of the high-affinity short-chain fatty acid/monocarboxylate transporter *SMCT1* is intriguing and requires further investigation.

## 1. Introduction

Obesity is associated with excess macronutrient (carbohydrates, fats, and proteins) intake and concomitant elevations in circulating amino acids, fatty acids, glucose and related metabolites [1,2]. Nutrient transporters (NT) play a key role in nutrient absorption and contribute to the regulation of circulating amino acids, fatty acids, glucose, and related metabolites. In *C. elegans*, loss of the peptide NT PEPT-1 results in higher uptake of free fatty acids and accumulation of body fat due to an increase in [H^+^] on the mucosal luminal surface [3]; conversely, loss of the sodium-proton exchanger NHX-2 leads to lower uptake of free fatty acids and reductions in body fat due to a decrease in [H^+^] on the mucosal luminal surface [3]. Moreover, compared to lean littermates, obese Zucker rats demonstrate higher mRNA and protein abundance for the amino acid NT *B^0^AT1* across all regions of the small intestine [4]. Obesity, as well as diabetes-related alterations in gastric emptying, may also affect nutrient absorption. While gastric emptying may be an important variable affecting nutrient absorption, the number and activity of NTs in the mucosa may also affect the ability of the mucosa to absorb exogenous nutrients. However, the impact that obesity and type 2 diabetes have on gastric emptying remains controversial [5,6,7]. Little is known regarding how the human intestine adapts to obesity [8] and fewer data exist examining the relationship between NTs and morbid obesity in humans.

The purpose of the present study was to evaluate the relationship between body mass index (BMI) and gene expression of NTs in a larger sample of severely obese patients undergoing Roux-En-Y Gastric Bypass (RYGB), as well as to examine a more diverse range of NTs. Specifically, we evaluated expression of 14 NTs (10 for amino acids, two for short-chain fatty acids, two for glucose) in relation to BMI. We hypothesized that expression of NTs associated with amino acids, short-chain fatty acids and monocarboxylates, and glucose would be positively correlated to BMI. Pre-operative serum samples were analyzed for branched and aromatic amino acids and short-chain fatty acids to determine correlations with their respective NTs. A more thorough understanding of the potential role that jejunal NT play in the regulation of BMI and associated alterations in circulating metabolites could provide novel targets for the development of future therapeutics.

## 2. Materials and Methods

### 2.1. Design and Setting

This cross-sectional study includes adult (>18 years of age) patients who underwent a Roux-en-Y gastric bypass surgery at Geisinger Medical Center (Danville, PA, USA). All participants in the present study are participating in an ongoing Bariatric Surgery cohort study approved by the Geisinger Institutional Review Board (IRB) [9]. All eligible participants provided IRB-approved, informed, written consent prior to their participation. All study participants met the National Institute of Health’s eligibility criteria for primary bariatric surgery (i.e., BMI ≥40 or >35 with qualifying comorbidities) [10]. Roux-en-Y candidates undergoing revision surgeries were excluded from the present study. Moreover, to receive Roux-en-Y at Geisinger Medical Center, all patients must be tobacco free for at least 2 months prior to their surgery [9]. All participants completed a standardized multidisciplinary preoperative program that included medical, psychological, nutritional, and surgical education as previously described [9]. As part of their standard of care preoperative clinic visits, patients’ height and weight were assessed in light-weight clothing in the absence of shoes and their waist circumference, at the level of the umbilicus, was assessed in the standing position using a non-elastic, flexible tape measure by trained nurses. BMI was calculated as weight in kg divided by height in m^2^. As part of the preoperative standard of care, all participants also received a liquid diet (~1000 kcal/day, 45% carbohydrate, 23% fat, and 32% protein) for two weeks prior to their surgery. Finally, the present study presentation is consistent with the STrengthening the Reporting of OBservational studies in Epidemiology (STROBE) statement for cross-sectional studies.

### 2.2. Participant Demographics and Clinical Characteristics

Forty-two patients undergoing primary RYGB participated in the study (Table 1). Sixteen participants were on metformin, four were on insulin, three were on sulfonylureas, and three were on other insulin sensitizers. Eighteen participants (43%) had diagnosed hypertension, of which five were on a single hypertensive medication and 13 were on two or more hypertensive medications. The most common hypertensive medications were beta blockers (*n* = 10), angiotensin converting enzyme inhibitors (*n* = 8), and loop diuretics (*n* = 8).

### 2.3. Jejunal Samples

Intraoperative proximal jejunal samples were obtained from 42 patients using jejunal tissue that would normally be discarded at the time of gastrojejunostomy or jejunojejunostomy construction. The mucosa was scraped on ice and immediately frozen in liquid nitrogen. Two µg of total RNA that was extracted using TRIzol (Invitrogen, Carlsbad, CA, USA) was used for cDNA synthesis using High Capacity cDNA Reverse Transcription kit (Applied Biosystems, Foster, CA, USA). Quantitative RT–PCR was performed with power SYBR Green PCR Master mix dye using StepOnePlus™ (Applied Biosystems) to measure the *C_T_* values for the target genes of interest as well as the CT values of the internal control gene (18S ribosomal RNA). Subsequently, we calculated the mRNA abundance using 40-Δ*C_T_* values (where Δ*C_T_* = target gene *C_T_* = internal control gene *C_T_*) to facilitate interpretation by aligning the direction of association so that higher values represent higher mRNA abundance. The information of the location of the NTs (i.e., apical vs. basolateral) in the mucosal cells was based on their established locations described in the literature [11]. The jejunal NTs that were investigated represent the transporters for the major macronutrients (glucose and amino acids). Supplementary Table S1 presents the primers used for mRNA quantification.

### 2.4. Glucose, Insulin, and HbA1c

Standard of care pre-operative blood tests were taken for glucose, insulin and HbA1c levels. These pre-operative clinical laboratory measurements were obtained from the (IRB approved) bariatric surgery research database housed in the Geisinger Obesity Institute. The Homeostatic Model Assessment of Insulin Resistance (HOMA-IR), a surrogate measure of insulin resistance, was calculated as glucose (mg/dL) × insulin (μU/mL)/405 [12].

### 2.5. Serum Amino Acid and Short-Chain Fatty Acid Analyses

In a subset of patients (*n* = 30) with overnight fasted, pre-operative, serum that were stored at −80 °C, we measured serum concentrations of branched-chain amino acids (leucine, isoleucine, and valine) and aromatic amino acids (phenylalanine and tyrosine) by liquid chromatography (LC) mass spectrometry as previously described [13] and concentrations of short-chain fatty acids (isobutyric acid, butyric acid, isovaleric acid, valeric acid, isocaproic acid, and hexanoic acid) by gas chromatography (GC) mass spectrometry as previously described [14]. All measures for isobutyric acid were above the standard curve; due to sample constraints, we were unable to re-run a diluted sample to obtain these values, and therefore we excluded these data from the present analysis.

### 2.6. Statistical Analyses

Correlations between pre-operative BMI and the mRNA abundance for NTs (using their 40-Δ*C_T_* values) were evaluated using Pearson’s correlations. In addition, linear trends between the pre-operative BMI groups and the mRNA abundance for the NT were evaluated using regression based analyses with and without adjustment for diabetes. Similar analyses were performed between pre-operative BMI and the pre-operative serum concentrations for branched-chain amino acids and short-chain fatty acids. Significance levels were *p* < 0.05. Data are presented as mean (standard deviation) [range].

## 3. Results

### 3.1. Jejunal Amino Acid Transporters

**Figure 1:**
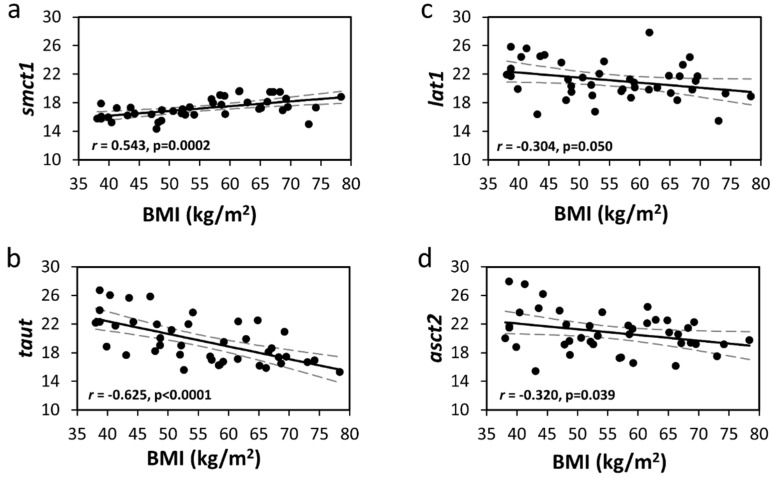
Correlations between the mRNA abundance of the nutrient transporters (NTs) *SMCT1* (**a**), *TauT* (**b**), *LAT1* (**c**), and *ASCT2* (**d**) with body mass index (BMI, kg/m^2^) in 42 severely obese patients undergoing a Roux-en-Y Gastric Bypass. The jejunal mucosa was scraped and flash-frozen in liquid nitrogen and stored at −80 °C. RNA was extracted from the samples and quantitative real-time–PCR performed for the NTs was studied. (*r* = Pearson’s correlation coefficient, solid line is the best fit regression line, slashed line is the 95% confidence interval). The mRNA abundance for the NTs are expressed as 40-Δ*C_T_*.

BMI negatively correlated with the mRNA abundance of the brush-border amino acid NTs *TauT* (*r* = −0.63, *p* < 0.0001, Table 2 and Figure 1) and *ASCT2* (*r* = −0.32, *p* = 0.039, Figure 1 and Table 2). There were also significant negative linear trends between the BMI categories and the mRNA abundance for *TauT* and *ASCT2*, independent of diabetes (Table 2). Likewise, waist circumference also negatively correlated with the mRNA abundance for *TauT* and *ASCT2* (Table 3). The negative correlation between BMI and the mRNA abundance of the basolateral amino acid NT *LAT1* also approached statistical significance (*r* = −0.30, *p* = 0.05, Figure 1 and Table 2). Moreover, there was also a significant negative linear trend between the BMI categories and the mRNA abundance for *LAT1* (Table 2). The mRNA abundance for the amino acid NTs *SNAT2*, *LAT2*, *CAT1*, *ATB*^0,+^, *B*^0^*AT1*, *xCT*, and *EAAT3* were not statistically associated with BMI (all *p* > 0.05, Table 2).

### 3.2. Jejunal Glucose and Short-Chain Fatty Acid/Monocarboxylate Transporters

BMI correlated positively with the mRNA abundance for the high-affinity short-chain fatty acid/monocarboxylate transporter *SMCT1* (*r* = 0.54, *p* = 0.0002, Figure 1 and Table 2). Moreover, there was also a significant positive linear trend between the BMI categories and the mRNA abundance for *SMCT1* (Table 2)*.* Waist circumference also positively correlated with the mRNA abundance for *SMCT1* (Table 3). BMI was not significantly correlated with the mRNA abundance for the glucose transporters *SGLT1* and *GLUT2* or the low-affinity short-chain fatty acid/monocarboxylate transporter *SMCT2* (*p* > 0.05, Table 2).

### 3.3. Pre-Operative Glucose, Insulin, HOMA-IR, and HbA1c

There were no statistically significant correlations between intestinal transporters and glucose, insulin, HOMA-IR, or HbA1c levels (*p* > 0.05, Table 3). In a diabetic subgroup analysis, these associations were consistent between groups.

### 3.4. Pre-Operative Serum Concentrations of Branched-Chain Amino Acids (BCAA) and Aromatic Amino Acids (AAA) 

There were no statistically significant associations between either the serum concentrations of the BCAA or AAA with BMI (*n* = 30) (all *p* > 0.05) (Table 4). Likewise, there were no significant associations between the serum concentrations of BCAA or AAA with the mRNA abundance for the amino acid NTs (*n* = 30) (all *p* > 0.05) (data not shown).

### 3.5. Pre-Operative Serum Concentrations of Short-Chain Fatty Acids

The serum concentrations of butyric, valeric, isocaproic acids correlated positively with BMI (*n* = 30) (Table 4). There were also positive linear trends between BMI categories and the serum concentrations of butyric and valeric acids. In addition, the serum concentrations of butyric and valeric acid correlated positively with the mRNA abundance for the high-affinity short-chain fatty acid/monocarboxylate transporter *SMCT1* in the jejunum (*n* = 30) (butyric acid: *r* = 0.40, *p* = 0.03; valeric acid: *r* = 0.37, *p* = 0.046).

## 4. Discussion

The principal finding in this study was that BMI correlated negatively with the mRNA abundance for three amino acid NTs (*TauT*, *ASCT2*, and *LAT1*) in the jejunum from patients with morbid obesity undergoing a Roux-en-Y gastric bypass, and positively correlated mRNA abundance for the high-affinity short-chain fatty/monocarboxylate acid transporter *SMCT1*. We also observed positive correlations between BMI and preoperative serum concentrations of several of the short-chain fatty acids including butyric, valeric, and isocaproic acid. These associations were independent of the presence of diabetes. Finally, we detected a positive correlation between the preoperative serum concentrations of several short-chain fatty acids and the mRNA abundance for the high-affinity short-chain fatty acid/monocarboxylate transporter *SMCT1* in the jejunum.

An important finding of the present study was the observation that preoperative BMI negatively correlated with the mRNA abundance for the amino acid NTs (*TauT*, *ASCT2*, and *LAT1*) in patients with morbid obesity undergoing bariatric surgery independent of the presence of diabetes. Moreover, elevated waist circumference was also negatively correlated with the jejunal mRNA abundance for *TauT* and *ASCT2* at the time of surgery. Although, the correlations between the mRNA abundance for *TauT*, *ASCT2*, and *LAT1* and BMI were in the low-to-moderate range, lower mRNA abundance of these amino acid NTs in patients with higher levels of obesity could have several implications. Lower abundance of amino acid NTs may protect against further elevations in circulating amino acids and amino acid metabolites associated with obesity and obesity-related insulin resistance [1]. Indeed, *TauT* and *ASCT2* are involved in the intestinal transport of alanine, serine, cysteine, threonine, glutamine, taurine, and β-alanine [15]. Moreover, *LAT1*, which negatively correlated with BMI in the present study (*p* = 0.05), regulates the intestinal transport of leucine, isoleucine, valine, phenylalanine, tyrosine, histidine and methionine [15]. Interestingly, however, the preoperative serum concentrations of the BCAA and AAA did not correlate with either BMI or the mRNA abundance for the amino acid NTs in these patients undergoing RYGB. There were also no statistically significant associations between the mRNA abundance for *TauT*, *ASCT2*, and the preoperative serum concentrations of amino acids known to be taken-up by these NT (data not shown). The underlying mechanism behind the negative correlation between BMI and these amino acid NTs remains unknown. However, we speculate that the excess caloric intake associated with higher BMI may be a primary mediator driving the down-regulation of these amino acid NTs. In addition, obesity is also related to elevations in circulating leptin concentrations, which could alter the expression of these amino acid NTs in the jejunum. For example, recent in vitro evidence indicates that leptin is associated with a down-regulation of amino acid transporters including *ASCT2* in human intestinal cells [16].

Another novel finding of the present study was the moderate positive correlation between the jejunal mRNA abundance for the high-affinity short-chain fatty acid/monocarboxylate NT *SMCT1* and BMI at the time of surgery. Moreover, elevated waist circumference was also positively correlated with the mRNA abundance for this transporter. *SMCT1* is best known for transporting the short-chain fatty acid butyrate in the colon [17]. However, *SMCT1’s* role in the jejunum remains unknown. Elevated mRNA abundance for *SMCT1* could lead to concomitant elevations in intestinal short-chain fatty acid uptake. Although a recent study has suggested that there is no difference in colonic uptake of short-chain fatty acids between lean and overweight adults [18], little is known about the uptake of short-chain fatty acids in the jejunum. Prior research has identified short-chain fatty acids, especially butyrate, as possessing important anti-inflammatory properties for the intestinal epithelium [19]. Moreover, the probiotic *Lactobacillus plantarum* increases the expression of *SMCT1* and counteracts the inflammatory effect of TNFα, in part by increasing butyrate uptake [17]. Future studies that assess the impact that BMI has on the mRNA, protein abundance, and function of these NTs will provide further clarification of their relationship. Moreover, additional studies to examine, to further elucidate the role of *SMCT1* in the uptake of short-chain fatty acids and other monocarboxylates in the jejunum, are needed. This is particularly important as the primary source of short-chain fatty acids occurs from the fermentation of dietary fiber in the colon [20].

We also observed positive correlations between serum short-chain fatty acid concentrations (butyric, valeric, and isocaproic acids) and BMI. The reason for the higher concentrations of these short-chain fatty acids in patients with higher BMI is not entirely clear. One plausible mechanism is an elevated uptake of short-chain fatty acids in the colon due, in part, to the elevated nutrient intake. Prior investigations have also reported that obesity is associated with elevations in the “leakiness” of the gastrointestinal tract [21,22], which could lead to elevated uptake of short-chain fatty acids through passive uptake, bypassing the short-chain fatty acid transporters altogether. Interestingly, the higher serum concentrations of the short-chain fatty acids were positively correlated with the mRNA abundance of the short-chain fatty acid transporter *SMCT1* in the jejunum even though the jejunum is not the primary site of short-chain fatty acid uptake [23]. Presently, whether the expression of *SMCT1* in the colon is altered in association with changes in BMI is unknown.

Here, we found no association between BMI and the glucose transporters *SGLT1* and *GLUT2* or the low-affinity short-chain fatty acid/monocarboxylate transporter *SMCT2* in these patients with morbid obesity undergoing a Roux-En-Y gastric bypass. Additionally, we also did not find any association between these transporters and pre-operative insulin, glucose, HOMA-IR, or HbA1c levels. Similarly, a previous study [24] also reported no association between *GLUT2* and BMI in bariatric surgery patients despite different sample sizes (*n* = 42 vs. *n* = 10) and BMIs (55.5 vs. 44.7 kg/m^2^). Collectively, our findings indicate that expression of *SGLT1*, *GLUT2*, and *SMCT2* may not depend on body mass, or vice versa, in patients with morbid obesity. Based on the present results, we cannot rule out an association between body mass and the glucose and Na-coupled low-affinity monocarboxylate transporters as people transition from normal weight, to obese, to severely obese. However, prior studies have indicated some differences worth noting between healthy lean, healthy obese, and morbidly obese patients. For example, a prior study revealed a higher expression of *GLUT2* and *SGLT1* in morbidly obese patients undergoing a Roux-en-Y gastric bypass compared to healthy normal weight people [25]. However, the biopsies in the controls were acquired from the distal duodenum, whereas in the morbidly obese patients the biopsies were acquired from the jejunum [25]. Moreover, the same study reported that bariatric surgery increased the expression of *GLUT2* and *SGLT1*, perhaps in an attempt to reduce carbohydrate malabsorption [25]. In another study, the duodenal expression of *SGLT1* was higher in patients with morbid obesity compared to healthy controls and was associated with higher glucose absorption and incretin levels [26].

The present study has several strengths and limitations. To date, this study is the most comprehensive evaluation of jejunal mRNA abundance for NTs (*n* = 14 transporters) in relation to BMI in a cohort of patients with morbid obesity (BMI: 38.1–78.4 kg/m^2^) undergoing a Roux-En-Y gastric bypass. All patients were on a liquid diet for two weeks prior to their surgery, which can be viewed as both a strength and a limitation of the study. Although the two-week liquid diet could reduce the variability in the mRNA abundance of the NTs, the liquid diet could also have direct effects on the expression levels of the jejunal NT that were collected at the time of surgery. For the first time, our study reports the associations between short chain fatty acids (SCFA), BMI, and their jejunal transporter in a cohort of patients with morbid obesity undergoing bariatric surgery. Future studies should also investigate the associations between other physiologically relevant SCFA, including acetate and propionate that are less affected by colonocyte metabolism, BMI, and their jejunal transporters. Our participants, however, were predominantly Caucasian (98%) and female (90%), and therefore the generalizability of our findings to other obese populations is unknown. Larger studies with more diverse samples are needed to further clarify the relationship between BMI and jejunal NTs. One limitation of the present study was that we were unable to measure protein abundance of the jejunal NT due sample limitations. Therefore, future studies examining the impact that BMI has on the mRNA abundance, protein abundance, and transporter activity of these NTs across the entire gastro-intestinal tract will provide further clarification of their relationship. Due to the cross-sectional study design, we were not able to determine whether changes in BMI resulted in changes in the mRNA abundance for the NTs or vice versa. Another limitation to this cross-sectional analysis is that there is no healthy, non-obese control as a reference group. However, the main purpose of the study was to identify the impact that BMI has on the expression of NTs in a cohort of patients with morbid obesity. Jejunal tissue samples are difficult to obtain, thereby making the data obtained herein unique, and the analysis does not lend itself to addressing whether the expression of jejunal NTs differ between non-obese controls and patients with morbid obesity. Clearly, future studies are warranted to determine if the expression of jejunal NTs differs between non-obese controls and patients with morbid obesity, perhaps using Double Balloon Eneteroscopy [27]. However, the indications for Double Balloon Enteroscopy include gastro-intestinal bleeds of unknown origin, or suspected tumors or ulcers detected by computed tomography or magnetic resonance imaging.

## 5. Conclusions

In summary, BMI was negatively associated with jejunal mRNA abundance for several amino acid NTs (*TauT*, *ASCT2*, and *LAT1)* and positively correlated with the mRNA abundance for the high-affinity short-chain fatty acid/monocarboxylate transporter *SMCT1*. BMI was also positively correlated with serum concentrations of butyric, valeric, and isocaproic acids. In conclusion, our findings provide rationale for future studies on the expression and function of NTs in the jejunum and their potential role in regulating nutrient absorption and the development of obesity.

## Figures and Tables

**Table 1:** Pre-operative sample characteristics (*n* = 42) *.

Female, %	90% (*n* = 38)
White, %	98% (*n* = 41)
Age, years	42.6 (11.9) [20, 64]
Body Mass Index, kg/m^2^	55.5 (11.3) [38.1, 78.4]
Diabetes	29% (*n* = 12)
Glucose, mg/dL	100.1 (33.2) [72, 264]
Insulin, µU/mL	22.8 (13.1) [5, 58.9]
HbA1c, %	5.9 (0.7) [5.1, 8.3]
HOMA-IR,	6.04 (4.03) [1.16, 18.68]
Waist circumference, cm	143.8 (19.7) [103.5, 182.9]
Total cholesterol, mg/dL	173.7 ± 38.1 [93, 266]
LDL cholesterol, mg/dL	101.5 ± 30.0 [39, 178]
HDL cholesterol, mg/dL	49.0 ± 12.0 [30, 86]
Triglycerides, mg/dL	116.0 ± 55.9 [44, 350]
Systolic Blood Pressure, mmHg	131.9 ± 17.4 [108, 198]
Diastolic Blood Pressure, mmHg	76.1 ± 8.8 [60, 100]

Data presented as % (*n*) or mean (standard deviation) [range]. * Multiply insulin values by 6 to convert to picomoles per liter; glucose values by 0.05551 to convert to millimoles per liter; low-density lipoprotein (LDL) and high-density lipoprotein (HDL) cholesterol values by 0.0259 to convert to millimoles per liter; and triglyceride values by 0.01129 to convert to millimoles per liter. The Homeostatic Model Assessment of Insulin Resistance (HOMA-IR) was calculated as glucose (mg/dL) × insulin (μU/mL)/405. Glucose levels were available for 40 patients, insulin for 39 patients, HbA1c for 38 patients, HOMA-IR for 39 patients, and waist circumference for 41 patients.

**Table 2:** The jejunal mRNA abundances of the nutrient transporters (NTs) in 42 severely obese patients undergoing a Roux-en-Y Gastric Bypass stratified by body mass index (kg/m^2^). The jejunal mucosa was scraped and immediately frozen in liquid nitrogen and stored at −80 °C. RNA was extracted from the samples and quantitative real-time–PCR was performed to determine the mRNA abundance of the NTs. The mRNA abundance for the NTs are expressed as 40-Δ*C_T_* values.

		NT mRNA Abundance (40-Δ*C_T_* Values) by BMI Quartile						Pearson’s Correlation	
	Overall *n* = 42	35–44 *n* = 10	45–54 *n* = 11	55–64 *n* = 10	65+ *n* = 11	*p*-value1	*p*-value2	*r*	*p*-value
*GLUT2*	22.7 (2.8)	23.2 (3.3)	21.5 (3.7)	23.8 (1.5)	22.4 (2)	0.957	0.856	–0.082	0.604
*SGLT1*	25.2 (3.3)	25.1 (4.6)	24.4 (3.3)	26.9 (1.7)	24.6 (2.9)	0.834	0.927	–0.066	0.676
*SMCT1*	17.2 (1.4)	16.4 (0.8)	16.2 (0.9)	18.3 (1.1)	18.0 (1.4)	<0.0001	<0.0001	0.543	0.0002
*SMCT2*	21.2 (3.1)	21.6 (3.7)	20.0 (3.4)	22.6 (2.8)	20.6 (2.3)	0.908	0.791	–0.080	0.616
*TauT*	19.7 (3.2)	22.7 (3.0)	20.4 (2.9)	18.5 (2.4)	17.3 (1.5)	<0.0001	<0.0001	–0.625	<0.0001
*ATB*^0,+^	19.1 (2.3)	18.4 (2.9)	18.4 (1.8)	20.0 (2.4)	19.4 (2.0)	0.175	0.204	0.183	0.245
*B*^0^*AT1*	23.7 (3.4)	23.4 (5.1)	22.9 (3.4)	25.4 (1.6)	23.2 (2.6)	0.695	0.785	–0.043	0.786
*SNAT2*	21.7 (2.6)	22.8 (3.0)	20.5 (2.8)	22.8 (1.3)	20.7 (2.2)	0.244	0.229	–0.229	0.144
*LAT1*	21.1 (2.6)	22.8 (2.9)	20.6 (2.2)	21.0 (2.6)	20.3 (2.5)	0.052	0.046	–0.304	0.050
*LAT2*	21.9 (2.6)	23.2 (2.8)	20.9 (2.9)	23.0 (1.6)	20.8 (2.4)	0.161	0.134	–0.278	0.074
*xCT*	18.7 (2.9)	19.8 (3.4)	17.1 (3.3)	19.3 (2.3)	18.9 (2.2)	0.881	0.846	–0.050	0.749
*CAT1*	19.7 (2.7)	21.0 (3.1)	18.6 (3.1)	20.6 (1.8)	19.0 (2.0)	0.256	0.217	–0.212	0.178
*EAAT3*	21.4 (2.8)	22.2 (3.1)	20.2 (3.1)	22.6 (1.8)	20.6 (2.6)	0.522	0.434	–0.172	0.275
*ASCT2*	20.8 (2.8)	22.7 (4.0)	20.6 (2.0)	20.6 (2.7)	19.6 (1.7)	0.018	0.016	–0.320	0.039

Data presented as mean (standard deviation); *p*-value1 = linear trend without adjustment; *p*-value2 = linear trend with adjustment for diabetes. *GLUT2*, glucose transporter 2 (*SLC2A2*); *SGLT1*, sodium-coupled glucose transporter 1 (*SLC5A1*); *SMCT1*, sodium-coupled monocarboxylate transporter 1 (*SLC5A8*); *SMCT2*, sodium-coupled monocarboxylate transporter 2 (*SLC5A12*); *TauT*, taurine transporter (*SLC6A6*); *ATB*^0,+^, amino acid transporter *B*^0,+^ (*SLC6A14*); *B*^0^*AT1*, neutral amino acid transporter (*SLC6A19*); *SNAT2*, sodium-dependent neutral amino acid transporter-2; *LAT1*, L-type amino acid transporter 1 (*SLC7A5*); *LAT2*, L-type amino acid transporter 2 (*SLC7A8*); *xCT*, catalytic subunit of the amino acid transporter x_c_ (*SLC7A11*); *CAT1*, cationic amino acid transporter 1 (*SLC7A1*); *EAAT3*, excitatory amino acid transporter 3 (*SLC1A1*); *ASCT2*, *ASC* amino acid transporter 2 (*SLC1A5*).

**Table 3:** Correlation between jejunal mRNA abundance of the nutrient transporters (NTs) glucose, insulin, HbA1c, HOMA-IR, and Waist Circumference. The mRNA abundance for the NTs are expressed as 40-Δ*C_T_* values *.

	Glucose		Insulin		HbA1c		HOMA-IR		Waist Circumference	
	*r*	*p*-value	*r*	*p*-value	*r*	*p*-value	*r*	*p*-value	*r*	*p*-value
*GLUT2*	0.193	0.234	0.160	0.330	0.188	0.257	0.212	0.196	–0.018	0.910
*SGLT1*	0.174	0.284	0.173	0.292	0.154	0.354	0.227	0.165	–0.013	0.935
*SMCT1*	–0.126	0.440	–0.239	0.143	–0.232	0.162	–0.296	0.068	0.476	0.0017
*SMCT2*	0.137	0.399	0.145	0.377	0.177	0.289	0.198	0.227	–0.021	0.895
*TauT*	0.238	0.139	0.079	0.633	0.219	0.186	0.206	0.208	–0.509	0.0007
*ATB*^0,+^	–0.002	0.989	0.163	0.320	0.019	0.909	0.157	0.339	0.210	0.187
*B*^0^*AT1*	0.166	0.307	0.184	0.261	0.188	0.259	0.234	0.152	0.026	0.870
*SNAT2*	0.071	0.665	0.187	0.255	0.020	0.905	0.224	0.171	–0.237	0.135
*LAT1*	0.080	0.624	0.043	0.795	0.046	0.785	–0.006	0.969	–0.241	0.130
*LAT2*	0.145	0.371	0.171	0.299	0.086	0.608	0.219	0.181	–0.204	0.200
*xCT*	0.052	0.749	0.148	0.367	0.030	0.858	0.143	0.384	–0.113	0.481
*CAT1*	0.121	0.455	0.150	0.362	0.046	0.786	0.164	0.318	–0.226	0.155
*EAAT3*	0.181	0.265	0.192	0.243	0.140	0.402	0.234	0.152	–0.147	0.358
*ASCT2*	0.030	0.856	0.028	0.866	–0.024	0.888	–0.006	0.970	–0.335	0.032

* Glucose levels were available for 40 patients, insulin for 39 patients, HbA1c for 38 patients, HOMA for 39 patients, and waist circumference for 41 patients.

**Table 4:** Association between preoperative concentrations of branched chain amino acids, aromatic amino acids, and short-chain fatty acids and BMI *.

		Mean Serum Metabolite Concentrations by BMI Quartile						Pearson’s Correlation	
	Overall *n* = 30	35–44 *n* = 7	45–54 *n* = 10	55–64 *n* = 7	65+ *n* = 6	*p*-value1	*p*-value2	*r*	*p*-value
Leucine, µmol/L	129.8 (24.3)	117.2 (25.1)	141.2 (27.4)	131.7 (17.1)	123.4 (20.9)	0.824	0.828	0.008	0.965
Isoleucine, µmol/L	61.7 (14.5)	58.1 (14.8)	66.7 (18.9)	63.3 (7.2)	55.7 (11.5)	0.677	0.678	–0.125	0.511
Valine, µmol/L	247.6 (45.2)	226.2 (56.1)	265.8 (44.1)	241.4 (25.3)	249.3 (49.4)	0.570	0.573	0.073	0.700
Phenylalanine, µmol/L	83.3 (15.9)	71.1 (4.6)	90.3 (19.2)	82.4 (17.9)	86.9 (7.7)	0.137	0.145	0.257	0.171
Tyrosine, µmol/L	66.2 (19.2)	64.0 (19.7)	75.5 (23.1)	54.9 (10.0)	66.5 (15.6)	0.692	0.692	–0.107	0.575
Butyric Acid, µmol/L	0.55 (0.21)	0.46 (0.10)	0.50 (0.24)	0.53 (0.13)	0.74 (0.23)	0.016	0.018	0.446	0.013
Isovaleric Acid, µmol/L	0.34 (0.24)	0.35 (0.22)	0.27 (0.11)	0.24 (0.10)	0.56 (0.41)	0.136	0.142	0.252	0.179
Valeric Acid, µmol/L	0.34 (0.13)	0.27 (0.07)	0.30 (0.12)	0.39 (0.16)	0.42 (0.12)	0.014	0.012	0.439	0.015
Isocaproic Acid, µmol/L	0.04 (0.04)	0.04 (0.03)	0.03 (0.02)	0.03 (0.02)	0.07 (0.06)	0.124	0.119	0.364	0.048
Hexanoic Acid, µmol/L	0.87 (0.34)	0.69 (0.15)	0.83 (0.26)	0.99 (0.57)	1.00 (0.22)	0.073	0.072	0.292	0.118

Data presented as mean (standard deviation). *p*-value1 = linear trend without adjustment. *p*-value2 = linear trend with adjustment for diabetes. * Serum samples were available for 30 patients for the above analyses.

## Supplementary Materials

The following are available online at http://www.mdpi.com/2072-6643/8/11/683/s1, Table S1: Primer sequences used for Real-Time Quantitative RT-PCR.
