# Supplementary material for: Nutrient Transporter Expression in the Jejunum in Relation to Body Mass Index in Patients Undergoing Bariatric Surgery
Source: Nutrients. 2016 Oct 29;8(11):683. doi: 10.3390/nu8110683 (PMC5133071; doi:10.3390/nu8110683)
Supplement: Supplementary file 1 [file nutrients-08-00683-s001.pdf]

Supplementary Materials: Nutrient Transporter Expression in the Jejunum in Relation to Body Mass Index in Patients Undergoing Bariatric Surgery

Brian A. Irving, G. Craig Wood, Peter N. Bennotti, Ellappan Babu, Abhishek Deshpande, Michelle R. Lent, Anthony Petrick, Jon Gabrielsen, William Strodel, Glenn S. Gerhard, Christopher D. Still, Vadivel Ganapathy and David D. K. Rolston

Table S1. Primer sequences used for Real-Time Quantitative RT-PCR.

Protein Name	Gene Name	Orientation	Sequence
GLUT2	SLC2A2	forward	GTGGGTGGCTTGGGGACACAC
GLUT2	SLC2A2	reverse	CCAGGCCTGAAATTAGCCCACAA
SGLT1	SLC5A1	forward	CCAAGACCACCGCGGTCACCC
SGLT1	SLC5A1	reverse	GAGGCTCCAATCGGCCACCAC
SMCT1	SLC5A8	forward	CGTCTCTGTGGAACAGTCCT
SMCT1	SLC5A8	reverse	TAAGACCACCCAGTGTGCAG
SMCT2	SLC5A12	forward	GGAGGTGTTGTGCAGGCTTCCC
SMCT2	SLC5A12	reverse	GGCCCCAATGGCCACCCAAA
TauT	SLC6A6	forward	CTTCTCTTGCTTGGACTGGATAG
TauT	SLC6A6	reverse	CTACACACGAAGGCGATGAA
$ATB^{0,+}$	SLC6A14	forward	GCAGCTTCCCAGTGAACAAT
$ATB^{0,+}$	SLC6A14	reverse	ATATACCACCTTGCCAGACG
$B^{0}AT1$	SLC6A19	forward	TCCATCCACCCGGCCCTGAAG
$B^{0}AT1$	SLC6A19	reverse	ACTCGTCCACATACCCTGTCTGGT
SNAT2	SLC38A2	forward	TGGGCAGTGGAATCCTTGGGC
SNAT2	SLC38A2	reverse	AAAGACCCTCCTTCATTGGCAGTCT
LAT1	SLC7A5	forward	CCGTGCCGTCCCTCGTGTTC
LAT1	SLC7A5	reverse	GGTTCACCTTGATGGGCCGCT
LAT2	SLC7A8	forward	TCGGCTCCTGGCTGCCATCT
LAT2	SLC7A8	reverse	CCAGCCAGAAGTACTCTCCTTTGC
xCT	SLC7A11	forward	TGGACGGTGTGTGGGGTCCT
xCT	SLC7A11	reverse	CAGCAGTAGCTGCAGGGCGTA
CAT-1	SLC7A1	forward	CTCGGGTGCCGTTGCTGCTGT
CAT-1	SLC7A1	reverse	CAGGTTAGGCTGCTCTGGCTGG
EAAT3,EAAC1	SLC1A1	forward	CCGCGGTGGTGCTAGGCATT
EAAT3,EAAC1	SLC1A1	reverse	ACGTTGGAATCCAGTGCAGCAA
ASCT2, AAAT	SLC1A5	forward	GCCATCAACGCCTCCGTGGGA
ASCT2, AAAT	SLC1A5	reverse	ACGGGCACCTTCACCCTGGTTC
18S		forward	CCCGTTGAACCCCATTCGT
18S		reverse	GCCTCACTAAACCATCCAATCGGTA
